# Impact of Preoperative Silodosin on Ureteroscopy Outcomes for Ureterolithiasis: A Systematic Review and Meta-Analysis

**DOI:** 10.1590/S1677-5538.IBJU.2025.0355

**Published:** 2025-10-30

**Authors:** Nathan Joseph Silva Godinho, Caio Hernandes Colhado, Lucas Guimarães Campos Roriz de Amorim, Marco Antonio Andrade, Marcos Antonio Dias Vilela, Samuel Elias Marinho da Costa, Thales Henrique Figueiredo Menezes, Michael Lipkin, Eduardo Mazzucchi

**Affiliations:** 1 Universidade Federal de Minas Gerais Departamento de Medicina Belo Horizonte MG Brasil Departamento de Medicina, Universidade Federal de Minas Gerais (UFMG), Belo Horizonte, MG, Brasil; 2 Faculdade de Medicina de São José do Rio Preto Departamento de Medicina São José do Rio Preto SP Brasil Departamento de Medicina, Faculdade de Medicina de São José do Rio Preto (FAMERP), São José do Rio Preto, SP, Brasil; 3 Universidade de São Paulo Departamento de Medicina São Paulo SP Brasil Departamento de Medicina, Universidade de São Paulo (USP), São Paulo, SP, Brasil; 4 Duke University Medical Center Department of Urology Durham NC United States Department of Urology, Duke University Medical Center, Durham, NC, United States; 5 Universidade de São Paulo Departamento de Cirurgia São Paulo SP Brasil Divisão de Urologia, Departamento de Cirurgia, Universidade de São Paulo (USP), São Paulo, SP, Brasil

**Keywords:** Adrenergic alpha-Antagonists, silodosin [Supplementary Concept], Meta-Analysis [Publication Type]

## Abstract

**Purpose:**

To perform a systematic review and meta-analysis evaluating the efficacy and safety of preoperative silodosin in improving ureteroscopy (URS) outcomes for ureterolithiasis.

**Materials and Methods:**

PubMed, EMBASE and Cochrane Central were systematically searched for studies comparing preoperative silodosin with placebo or ‘no preoperative silodosin’ in patients undergoing URS for ureteral stones. Primary outcomes included ureteral wall injury, analgesia use, fever, haematuria, stone-free rate (SFR), operative time, and complications. Statistical analysis was performed using Review Manager 5.1.7. Study quality and risk of bias were assessed per Cochrane guidelines.

**Results:**

Nine studies, including eight randomized clinical trials, including 960 patients were analysed; 450 (46.8%) received silodosin. Compared to controls, silodosin significantly reduced ureteral injuries (RR 0.30; 95% CI: 0.18–0.49; p < 0.00001) and operative time (MD −17.72 minutes; 95% CI: −24.72 to −10.72; p < 0.00001). It also lowered analgesia needs (RR 0.35; 95% CI: 0.16–0.75; p = 0.007), with trends toward reduced fever (RR 0.67; 95% CI: 0.36–1.22; p = 0.19) and haematuria (RR 0.57; 95% CI: 0.32–1.02; p = 0.06). In studies with ≥10 days of preoperative use, silodosin significantly improved SFR (RR 1.17; 95% CI: 1.10–1.26; p < 0.00001).

**Conclusions:**

Preoperative silodosin reduces ureteral injuries, operative time, and complications, supporting its use to improve safety and efficiency of URS for ureterolithiasis.

## INTRODUCTION

Ureterolithiasis, defined as the presence of calculi within the ureter, represents a common urological condition associated with significant clinical morbidity, including acute pain, urinary tract obstruction, and other complications necessitating timely intervention (1, 2). Ureteroscopy (URS) has emerged as a cornerstone modality for the management of ureteral stones, offering high stone-free rates and broad applicability. Despite its efficacy, URS is not without technical challenges; it is frequently associated with prolonged operative times, the need for ureteral dilation, and procedural complications that may adversely affect patient outcomes and recovery (1).

In an effort to address these challenges, pharmacological adjuncts, most notably α-adrenergic receptor antagonists, have been explored for their capacity to optimize preoperative conditions. Among these, silodosin, a highly selective α1A-adrenergic receptor blocker, has garnered increasing attention for its potential to improve ureteroscopic outcomes, particularly in comparison to tamsulosin in the context of distal ureteral calculi (3). Silodosin's greater selectivity for α1A receptors, as opposed to tamsulosin's broader affinity for both α1A and α1D subtypes, may enhance its efficacy in promoting ureteral smooth muscle relaxation and facilitating stone passage (3). These pharmacodynamic properties have led to the conduction of several randomized controlled trials (RCTs) evaluating silodosin's role in the preoperative setting.

Accordingly, we conducted a systematic review and meta-analysis to assess the impact of preoperative silodosin on the safety and efficacy of URS for ureterolithiasis. Specifically, this study evaluates outcomes including ureteral wall injury, stone-free rate (SFR), operative time, analgesic requirement, and perioperative complications. By synthesizing current evidence, it seeks to clarify silodosin's role in optimizing ureteroscopic procedures and to provide high-quality data to support clinical decision-making in urological practice.

## MATERIALS AND METHODS

### Protocol and Registration

This systematic review and meta-analysis followed the Cochrane Handbook and PRISMA guidelines (4, 5). The protocol was prospectively registered in the International Prospective Register of Systematic Reviews (PROSPERO) (protocol: CRD42025633316).

### Inclusion and Exclusion Criteria

Studies were included if they: (I) compared preoperative silodosin with a control; (II) involved patients undergoing ureteroscopy (URS); and (III) addressed ureterolithiasis. The control groups included no treatment or placebo, defined as an inert substance mimicking silodosin without pharmacologic effects. These comparators served to isolate silodosin's specific impact on surgical outcomes.

Conversely, studies were excluded if they were animal studies, case reports, or case series, as well as those that did not align with the PICOT framework. Specifically: (P) Population – patients with ureterolithiasis scheduled for URS; (I) Intervention – preoperative use of silodosin; (C) Comparison – no alpha-blockers or placebo; (O) Outcomes – intraoperative dilation, SFR, operative time, hospital stay, ureteral navigation, and complications; and (T) Type of studies – primary studies only, thereby excluding animal studies and case reports or series.

### Search Strategy

Searches were conducted in PubMed, Embase, and Cochrane databases for studies published between 2014 and 2024. No language or sample size restrictions were applied. The search strategy is detailed in Supplementary Table-S1 (see material supplementary).

### Study Selection and Data Extraction

Two reviewers independently screened studies using Rayyan software (6), resolving discrepancies by consensus. Data were extracted by one reviewer and cross-checked by the other. Extracted variables included study design, sample size, age, BMI, stone location, stone size, and outcomes. All data were stored in a standardized database.

### Endpoints and Definitions

The endpoints of interest were categorized as intraoperative and postoperative. Intraoperative endpoints included operative time, ureteral wall injury, and need for dilation (defined as requiring dilation if the ureteroscope could not pass the ureterovesical junction). Postoperative endpoints included SFR (residual fragments < 4 mm), need for analgesia, fever (≥ 38° C), and haematuria. Follow-up timing and imaging varied by study protocol. Only studies with comparable definitions were included in outcome-specific syntheses.

### Quality Assessment

Quality assessment of included studies was conducted using Cochrane tools.: RoB 2 for RCTs (7) and ROBINS-I for non-randomized studies (8), ensuring reliability and transparency of findings.

## Statistical analysis

Meta-analyses were conducted using Review Manager 5.4 (Copenhagen) (9). For dichotomous outcomes, risk ratios (RRs) with 95% confidence intervals (CIs) were calculated, whereas continuous outcomes were analysed using mean differences (MDs). Moreover, a random-effects model was employed, as variations in study populations and protocols were anticipated.

In addition, heterogeneity was assessed via Cochran's Q and I^2^ statistics, with p < 0.10 and I^2^ > 25% considered significant. To further address heterogeneity, sensitivity analyses were performed. Furthermore, subgroup analyses were conducted based on study type (RCT vs. non-RCT) and duration of silodosin use (<10 vs. ≥10 days). Finally, when only medians and interquartile ranges were reported, means and standard deviations were estimated using the method proposed by Wan et al. (10).

## RESULTS

### Selected Studies and Baseline Characteristics

**Figure 1:**
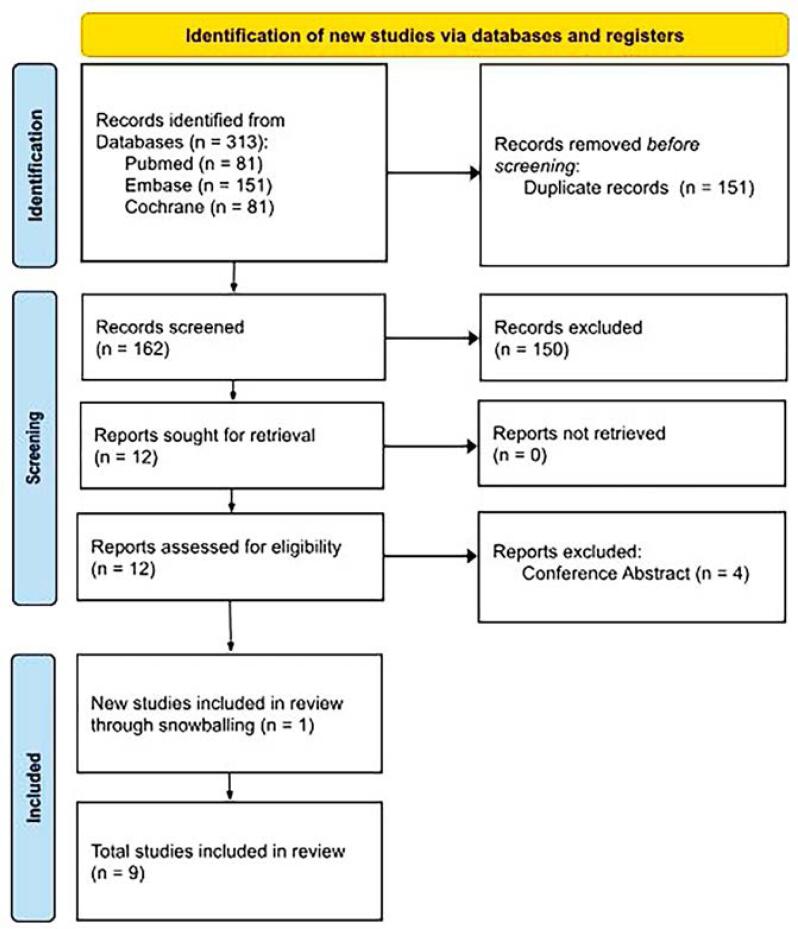
PRISMA flow diagram of study screening and selection.

A total of 313 articles were identified through PubMed, Embase, and Cochrane. After removing 151 duplicates, 162 records were screened, and 12 underwent full-text review. Four conference abstracts were excluded. Additional studies identified via backward snowballing brought the final number to nine included in the meta-analysis. The selection process is detailed in the PRISMA flow diagram (Figure-1), with the full checklist in the supplementary material (Figures S12–S14) (see material supplementary).

Nine studies (eight RCTs) with 960 patients were analysed (1, 2, 11–17). Of these, 450 (46.8%) received 8 mg/day of silodosin for 3–14 days before URS. Follow-up ranged from 1 to 3 months. Additionally, 613 patients were male (63.8%) and 145 (55.9%) had lower ureteral stones. Baseline characteristics are summarized in Table-1 and Table-S2 (see material supplementary).

**Table 1:** Baseline characteristics of the included studies.

Study, year	Study Design	Type of Control	Follow-up (months)	Time of therapy (dways)	Baseline Population Size, No. Silodosin Control	Age, years (mean ± SD) Silodosin Control	BMI, kg/mm² (mean ± SD) Silodosin Control	Male, No. (%) Silodosin Control	Stone size, mm (mean ± SD) Silodosin Control	Location of ureteral calculi (upper/top/lower) Silodosin Control
Alaridy et al., 2020 (2)	Non-RCT	Placebo	1	7	34 34	33.29 ± 9.51 34.60 ± 12.01	NA	25 (73.52) 25 (73.52)	10.35 ± 2.38 10.41 ± 2.43	3/6/25 3/6/25
Aydin et al., 2017 (11)	RCT	No pretreatment	1	3	47 5	43.00 ± 14.29* 37.50 ± 12.50*	NA	32 (68.08) 33 (66.00)	NA	12/9/26 8/12/30
Bhattar et al., 2017 (12)	RCT	Placebo	NA	14	23 21	35.52 ± 11.00 33.22 ± 10.07	23.34 34.10	15 (65.21) 15 (71.42)	9.14 ± 1.52 9.74 ± 1.98	5/4/14 6/3/12
Diab et al., 2023 (1)	RCT	Placebo	3	7	69 67	41.40 ± 14.26 42.40 ± 15.44	26.90 ± 3.79, 27.30 ± 3.97	38 (46.37) 41 (61.19)	12.50 ± 3.91 13.00 ± 3.71	70/0/0 70/0/0
Goyal et al., 2021 (13)	RCT	Placebo	0.5	10	84 141	39.28 ± 8.25 38.22 ± 8.34	27.75 ± 2.22 27.46 ± 2.29	53 (63.19) 86 (60.99)	8.77 ± 4.12 8.53 ± 0.49	0/0/84 0/0/93
Kim et al., 2021 (14)	RCT	No pretreatment	3	3	43 44	48.50 ± 11.60 45.80 ± 13.80	26.80± 4.90, 25.20 ± 3.30	29 (67.44) 23 (52.27)	8.86 ± 3.60 8.68 ± 5.07	50/0/0 50/0/0
Köprü et al., 2020 (15)	RCT	No pretreatment	3	10	38 38	45.41 ± 12.88* 46.52 ± 14.52*	NA	30 (78.94) 23 (60.52)	19.02 ± 5.90 17.94 ± 4.60	2/8/6 2/6/7
Mohey et al., 2018 (16)	RCT	Placebo	1	10	62 65	38.27 ± 9.37 39.67 ± 9.54	27.55 ± 2.28 27.80 ± 3.50	39 (62.90) 39 (60.00)	12.60 ± 1.25 12.90 ± 1.29	0/0/62 0/0/65
Shaher et al., 2023 (17)	RCT	Placebo	1	10	50 50	44.65 ± 10.13 45.37 ± 12.78	26.12 ± 2.63 26.34 ± 2.74	37 (74.00) 30 (60.00)	18.33 ± 5.17 17.61 ± 4.25	11/0/0 8/0/0

BMI = Body Mass Index; RCT = Randomised controlled trial; NA = Not available

* Mean and standard deviation (SD) estimated from median and interquartile range or median and range

### Quality Assessment

Two reviewers independently appraised the quality of individual studies. Notably, two RCTs raised some bias concerns: Aydin et al. (2), due to differences in ureteroscope use, and Goyal et al. (15), due to unclear blinding. Furthermore, Alaridy et al. (2) was rated as having moderate risk of bias per ROBINS-I, owing to unadjusted confounders and missing data.

### Endpoints Pooled Analysis

**Figure 2:**
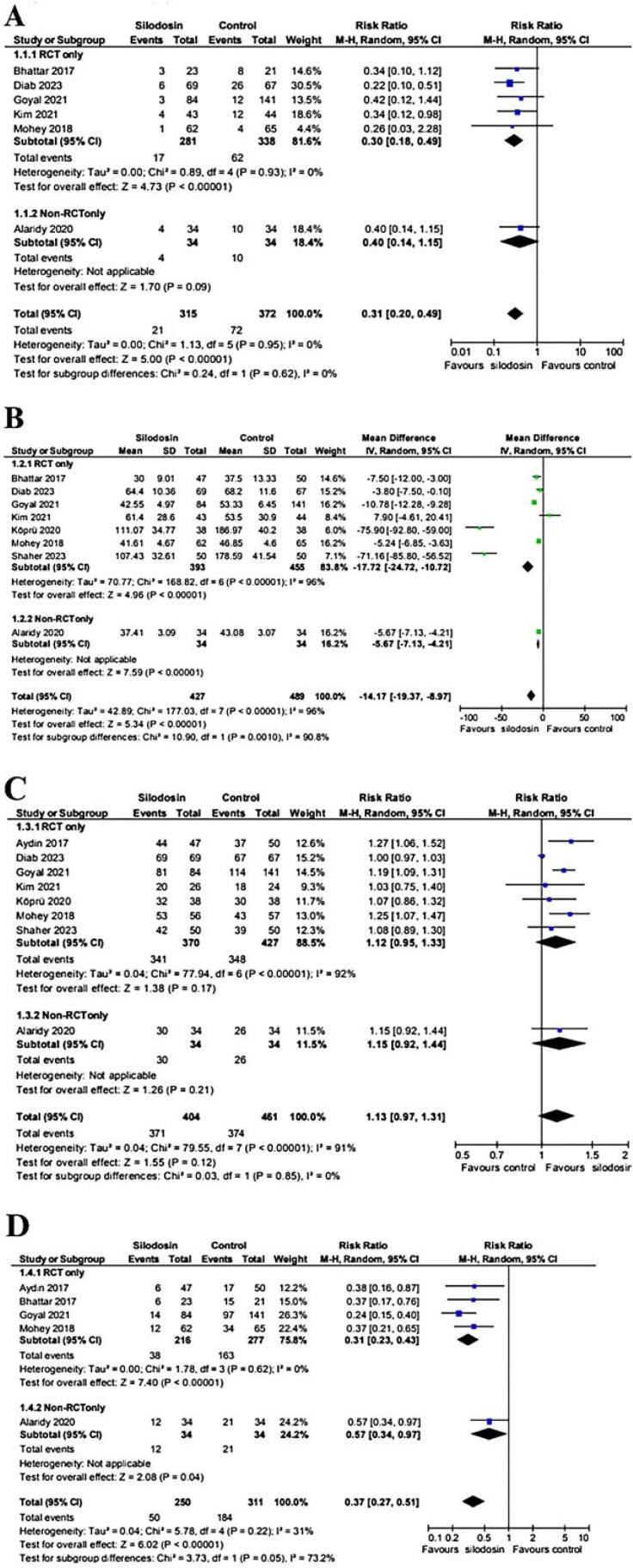
Forest plots for pooled risk ratio and mean difference of significant ureteral wall injury (A), operative time (B), SFR (C), and ureteral dilation required (D).

A meta-analysis showed that preoperative silodosin significantly improved 6 outcomes. It reduced ureteral injury (RR 0.31; 95% CI: 0.20–0.49; p < 0.00001; I^2^ = 0%; Figure-2A) and shortened operative time by 14.17 minutes (95% CI: −19.37 to −8.97; p < 0.000001; I^2^ = 96%; Figure-2B).

The SFR showed no significant difference between the silodosin and control groups (RR 1.13; 95% CI 0.97 – 1.31; p = 0.12; I^2^ = 91%; Figure-2C). However, it is important to note that the timing and method of postoperative imaging to assess stone-free status varied considerably across the included studies. Some trials performed evaluations as early as 1 week after surgery, whereas others waited up to 3 months. Additionally, the imaging modalities used were not standardized, further contributing to the observed heterogeneity. Despite these variations in follow-up protocols, the requirement for ureteral dilation was significantly lower in the silodosin group (RR 0.37; 95% CI 0.27 – 0.51; p < 0.00001; I^2^ = 31%; Figure-2D), and silodosin-treated patients required less postoperative analgesia than controls (RR 0.46; 95% CI 0.25–0.82; p = 0.009; I^2^ = 0%; Figure-S2) (see material supplementary).

### Subgroup Analyses

#### Subgroup Analysis of RCTs

In the subgroup analyses limited to RCTs, the previously observed outcomes remained consistent in both direction and statistical significance. The incidence of ureteral wall injury (RR 0.30; 95% CI 0.18 - 0.49; p < 0.00001; I^2^ = 0%; Figure-2A), reduction in operative time (MD −17.72; 95% CI −24.72 to −10.72; p < 0.00001; I^2^ = 96%; Figure-2B), lower requirement for ureteral dilation (RR 0.31; 95% CI 0.23 - 0.43; p < 0.00001; I^2^ = 0%; Figure-2D), reduced need for postoperative analgesia (RR 0.35; CI 0.16 - 0.75; p = 0.007; I^2^ = 0%; Figure-S2) (see material supplementary), as well as fewer cases of postoperative fever (RR 0.49; 95% CI 0.27 - 0.88; p = 0.02; I^2^ = 0%; Figure-S3A) (see material supplementary) and haematuria (RR 0.52; CI 0.28 - 0.98; p = 0.04; I^2^ = 0%; Figure-S3B) (see material supplementary) all continued to favour the silodosin group. The SFR remained statistically similar between the silodosin and control groups (RR 1.12; 95% CI 0.95 - 1.33; p = 0.17; I^2^ = 92%; Figure-2C). This consistency across RCTs strengthens the robustness of the findings and supports silodosin's effectiveness as a preoperative option for patients undergoing URS.

#### Subgroup Analysis Stratified by Duration of Preoperative Therapy (≥10 days vs <10 days)

In the subgroup analyses stratified into studies that conducted pre-URS therapy for ten days or more and those with therapy lasting fewer than ten days, the previously observed outcomes remained consistent in both direction and statistical significance (Figures S4, S5, and S6) (see material supplementary), except for the SFR outcome. A significant improvement in SFR was observed compared to the control in the subgroup receiving Silodosin for ≥ 10 days (RR 1.17, 95% CI: 1.10 – 1.26, p < 0.00001; I^2^ = 0; Figure-S7) (see material supplementary). In contrast, the subgroup with therapy duration < 10 days showed no significant difference (RR 1.11, 95% CI: 0.82 – 1.49, p = 0.48; I^2^ = 93; Figure-S5) (see material supplementary). Despite these findings, the test for subgroup differences revealed no statistically significant effect modification by therapy duration (p = 0.70).

#### Subgroup Analysis of Different Calculi Location

We performed a subgroup analysis stratifying data by stone location (distal ureteric stones, proximal ureteric stones, and studies including mixed locations) (Figures S8-S11) (see material supplementary).

The use of preoperative silodosin was associated with improved outcomes, particularly for distal ureteral calculi, whereas proximal stones generally showed non-significant results for several endpoints. This pattern was consistently observed across operative time, need for analgesia, and SFR.

Distal calculi treated with silodosin demonstrated a significant reduction in operative time (MD −8.02; 95% CI: –13.45 to –2.59; p = 0.004; I^2^ = 96%; Figure-S8) (see material supplementary), whereas proximal calculi showed a non-significant reduction (MD −21.92; 95% CI: –59.09 to 15.26; I^2^ = 98%; p = 0.25; Figure-S8). Silodosin significantly reduced the requirement for postoperative analgesia in distal stones (RR 0.31; 95% CI: 0.12–0.79; p = 0.01; Figure-S9) (see material supplementary), while no significant difference was observed for proximal (RR 0.45; 95% CI: 0.12–1.77; p = 0.25; I^2^ = 0%; Figure-S9). Distal calculi exhibited a significant improvement in SFR with silodosin (RR 1.21; 95% CI: 1.12–1.31; p < 0.00001; I^2^ = 0%; Figure-S11) (see material supplementary), as did mixed-location stones (RR 1.17; 95% CI: 1.04–1.32; p = 0.008; I^2^ = 0%; Figure-S11). Proximal stones showed no significant effect (RR 1.02; 95% CI 0.90–1.16; p = 0.73; I^2^ = 46%; Figure-S11).

Interestingly, silodosin significantly reduced wall injury rates for proximal calculi (RR 0.26; 95% CI: 0.14–0.50; p < 0.0001; I^2^ = 0%; Figure-S8) and mixed-location stones (RR 0.37; 95% CI 0.17–0.82; p = 0.01; I^2^ = 0%; Figure-S8), while a non-significant reduction was observed for distal stones (RR 0.37; 95% CI: 0.13–1.09; 0.07; I^2^ = 0%; Figure-S8).

Due to a lack of events, no pooled effect could be estimated for proximal calculi on the outcome of need for ureteral dilation. However, distal (RR 0.29; 95% CI: 0.19–0.44; p < 0.0001; I^2^ = 21%; Figure-S9) and mixed-location stones (RR 0.46; 95% CI 0.32–0.68; p < 0.0001; I^2^ = 0%; Figure-S9) showed consistent significant reductions.

When stratified by stone location, no significant differences were observed for either fever or haematuria (Figure-S10) (see material supplementary). However, pooled analysis across all locations revealed a significant reduction in postoperative fever (p = 0.02) and haematuria (p = 0.02) with preoperative silodosin.

### Sensitivity Analysis

We conducted leave-one-out sensitivity analyses to assess the robustness of our findings for outcomes with elevated heterogeneity. For ureteral wall injury, operative time, and ureteral dilation, the exclusion of individual studies did not impact the statistical significance or the I^2^ statistics. This confirms the consistency of the results and indicates they are not disproportionately influenced by any single study. However, the SFR, excluding the study by Diab et al. (13), resulted in a substantial change in effect size, favouring the silodosin group with a RR of 1.18 (95% CI 1.11 - 1.25; p < 0.00001). Moreover, the I^2^ statistic decreased dramatically from 91% to 0% upon the exclusion of this study. These findings highlight its significant impact on the overall results and suggest it was a major source of variability.

## DISCUSSION

This systematic review and meta-analysis demonstrated that preoperative silodosin improves both the safety and efficiency of ureteroscopy (URS) for ureterolithiasis. Specifically, silodosin significantly reduced ureteral wall injury, operative time, ureteral dilation, need for analgesia, fever, and haematuria.

Moreover, these findings align with those of Bhojani et al. (18), who showed that alpha-blockers benefit URS outcomes. However, their study evaluated the drug class as a whole, whereas ours focused specifically on silodosin. Notably, silodosin has shown superiority over tamsulosin, likely due to its higher α1A receptor selectivity (3).

Ureteral wall injury, a key endpoint in six studies, can cause serious complications such as avulsion (19). In this context, our analysis demonstrated consistent reductions in injury rates across subgroups and in sensitivity analyses. In addition, the reduced operative time observed in the silodosin group may reflect its ability to relax ureteral smooth muscle, thereby easing scope passage and decreasing the need for mechanical dilation (20).

Consequently, shorter surgeries may also explain the lower incidence of postoperative fever, as reduced tissue manipulation and trauma likely diminish the risk of infection. By facilitating smoother endoscope advancement, silodosin minimizes ureteral irritation, which may translate to fewer postoperative complications.

Regarding treatment duration, it ranged from 3 to 14 days across the included studies. Although all durations demonstrated some benefit, longer silodosin treatment was associated with significantly higher SFR, supported by low heterogeneity (I^2^ = 0%) and narrower confidence intervals. In contrast, the subgroup with <10 days of treatment showed no significant benefit and exhibited high heterogeneity. Although the difference between subgroups was not statistically significant, longer silodosin exposure may enhance ureteral relaxation and stone clearance, thus warranting further investigation.

Regarding stone location, preoperative silodosin significantly improved outcomes in ureteroscopy, particularly for distal ureteral stones, where reductions in operative time, analgesic requirement, and higher stone-free rates were observed. This is consistent with the known distribution of α1-adrenergic receptors, which are more densely expressed in the distal ureter (21). Proximal calculi did not show consistent benefits in efficiency but demonstrated a marked reduction in wall injury.

Furthermore, variability in surgical techniques, such as the use of rigid versus flexible ureteroscopes, access sheaths, and different laser technologies, may have influenced the observed outcomes. Institutional resources and surgeon experience likely contributed to these variations. Additionally, patient-related factors, including comorbidities and stone characteristics (size and location), may have added to the heterogeneity. While some studies focused on distal ureteral stones, where alpha-blockers are particularly effective (22, 23), others included stones at various ureteral locations. Regarding BMI, all included studies reported a mean BMI within the overweight range in both the silodosin and control groups. The only exception was one study (12), in which the mean BMI was in the normal range for the silodosin group, whereas the control group had a mean BMI in the class I obesity range. These discrepancies likely explain the heterogeneity in certain outcomes, despite subgroup and sensitivity analyses.

A key strength of this meta-analysis is its individualized assessment of each complication, thereby avoiding potential bias from composite outcome reporting. Indeed, grouping complications could lead to double-counting patients and obscure drug-specific effects. Our findings, therefore, support silodosin's favourable safety profile, showing reductions in complications and operative time. Although adverse events were not uniformly reported, existing data suggest that silodosin may be safer than other alpha-blockers such as tamsulosin (3, 24).

In conclusion, silodosin appears to be an effective and safe preoperative adjunct in URS. It reduces complications and operative time, with potential advantages for extended preoperative use. Nevertheless, heterogeneity across studies and inconsistent adverse event reporting underscore the need for standardized protocols and further high-quality trials to define its optimal clinical application.

### Limitations

This meta-analysis provides Level 1 evidence supporting preoperative silodosin use before URS for ureterolithiasis. However, several limitations must be acknowledged.

First, the stone location varied considerably among patients, potentially influencing procedural difficulty and outcomes. Second, significant heterogeneity was noted in the assessment of SFR, including inconsistent definitions (e.g., residual fragments < 2 mm vs. 0 mm), different imaging modalities (CT, X-ray, or ultrasound), and varied follow-up timing (1 week to 3 months). These inconsistencies limit the comparability of SFR results.

Third, none of the RCTs accounted for spontaneous stone expulsion rates, which may have reduced the true effect size in patients who might not have required surgery. Fourth, essential procedural variables, such as stone location, surgical technique, stent placement, and duration, were not uniformly reported across studies, potentially confounding analyses of postoperative outcomes like pain and hematuria, which may often be attributed to ureteric stent use and may not significantly impact patient management or outcomes after ureteroscopy.

Lastly, stricture formation, a relevant long-term complication, was not addressed in any of the included studies. This omission restricts the evaluation of silodosin's potential long-term protective effects.

These limitations underscore the challenge of synthesizing data from heterogeneous trials and highlight the need for future research employing standardized protocols, uniform definitions, and comprehensive outcome reporting to better define silodosin's role in URS optimization.

## CONCLUSIONS

In this meta-analysis, utilizing silodosin as a preoperative treatment in the URS approach for ureterolithiasis improves both the safety and efficiency of the procedure compared to no preoperative therapy. Future research should prioritize RCTs that incorporate stratification based on stone location while also focusing on standardizing the definition of SFR, ensuring proper follow-up, and optimizing preoperative silodosin treatment duration.

## Data Availability

All data generated or analysed during this study are included in this published article

## ABBREVIATIONS

BMI: = Body Mass Index
CI: = Confidence intervals
MD: = Mean difference
PICOT: = Population, intervention, comparison, outcome, and type of studies
PRISMA: = Preferred Reporting Items for Systematic Reviews and Meta-Analysis
PROSPERO: = Prospective Register of Systematic Reviews
RCT: = Randomized controlled trial
RoB 2: = Risk of Bias 2
ROBINS-I: = Risk of Bias in Non-Randomized Studies of Interventions
RR: = Risk ratio
SD: = Standard deviation
SFR: = Stone-free rate
URS: = Ureteroscopy
