# Supplementary material for: Impact of Preoperative Silodosin on Ureteroscopy Outcomes for Ureterolithiasis: A Systematic Review and Meta-Analysis
Source: Int Braz J Urol. 2025 Oct 30;52(2):e20250355. doi: 10.1590/S1677-5538.IBJU.2025.0355 (PMC13124197; doi:10.1590/S1677-5538.IBJU.2025.0355)
Supplement: Supplementary file 1 [file 1677-6119-ibju-52-02-e20250355-suppl1.pdf]

APPENDIX

SUPPLEMENTARY MATERIALS

Impact of Preoperative Silodosin on Ureteroscopy Outcomes for Ureterolithiasis: A Systematic Review and Meta-Analysis

SUPPLEMENTARY FIGURES

Figure S1 - Diagram of Risk of Bias assessment in randomised trials using the RoB 2 tool (A) and non-randomised trials using the ROBINS-I tool (B).

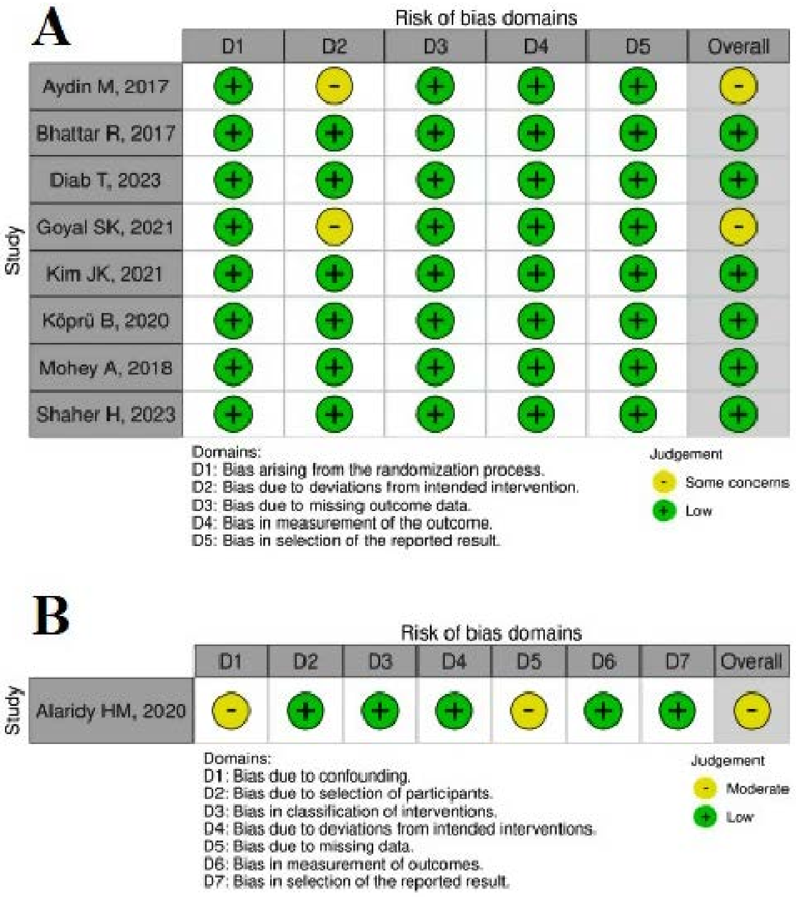

Figure S2 - Forrest plot: Need for analgesia.

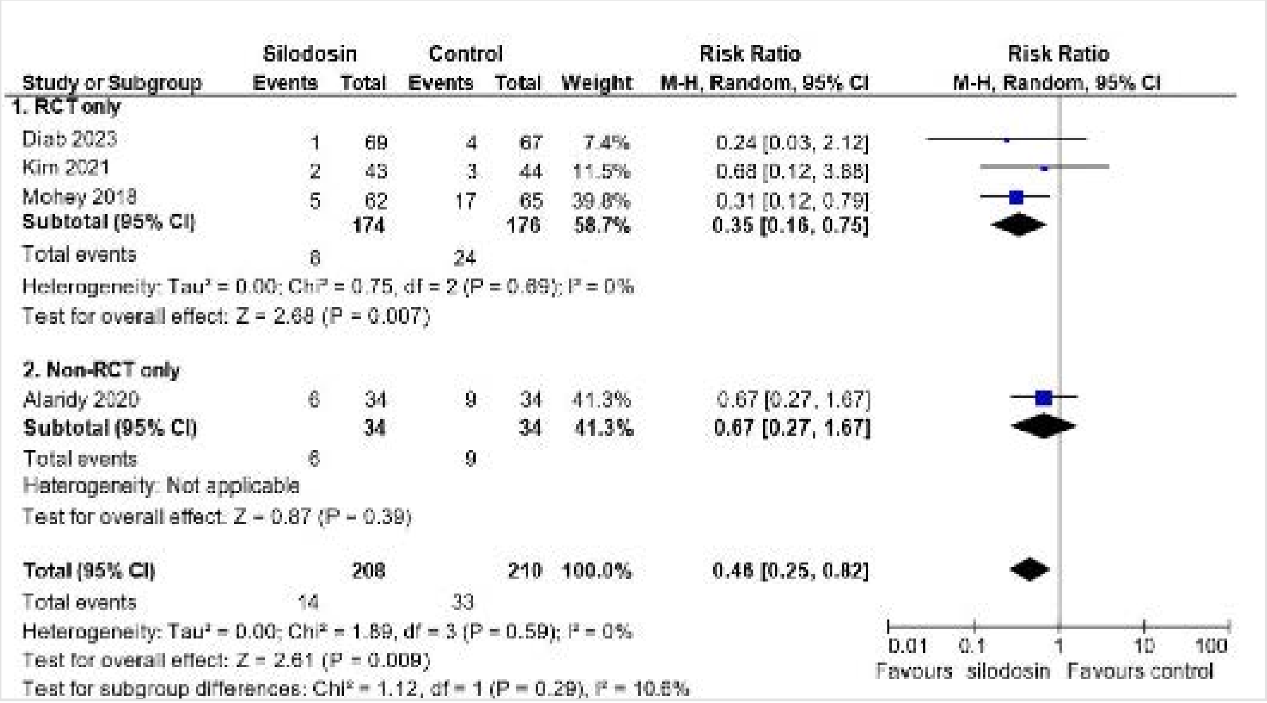

Figure S3 - Forrest plot: Post-operative fever (A) and hematuria (B).

A) Fever (post-operative)

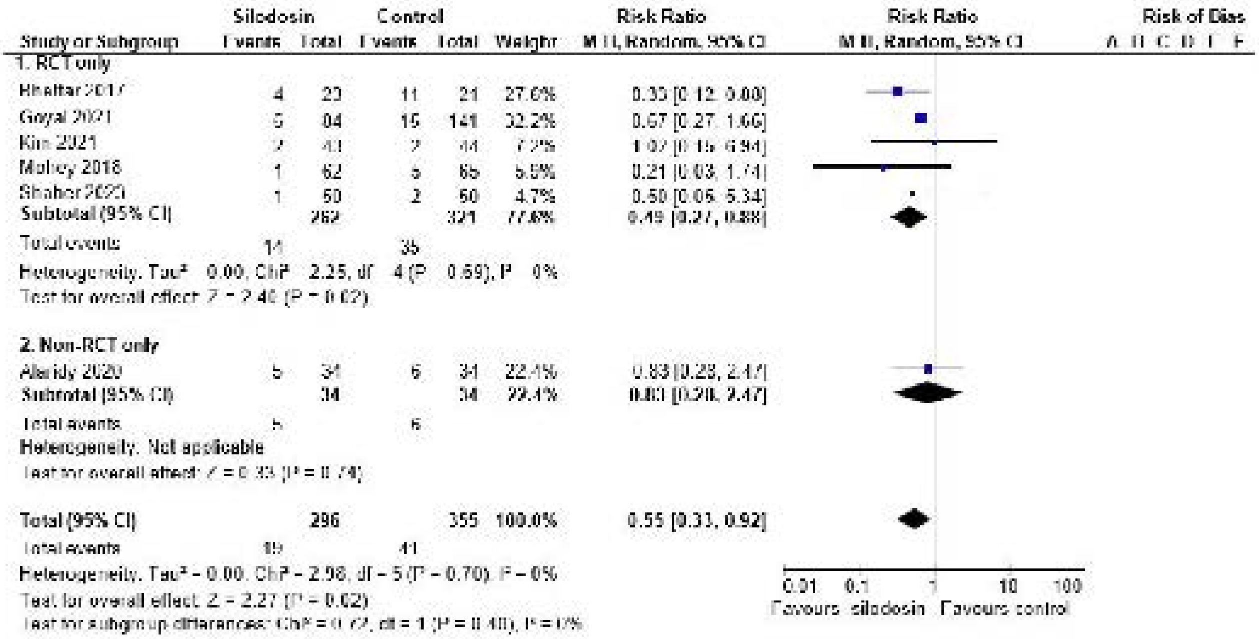

B) Haematuria (post-operative)

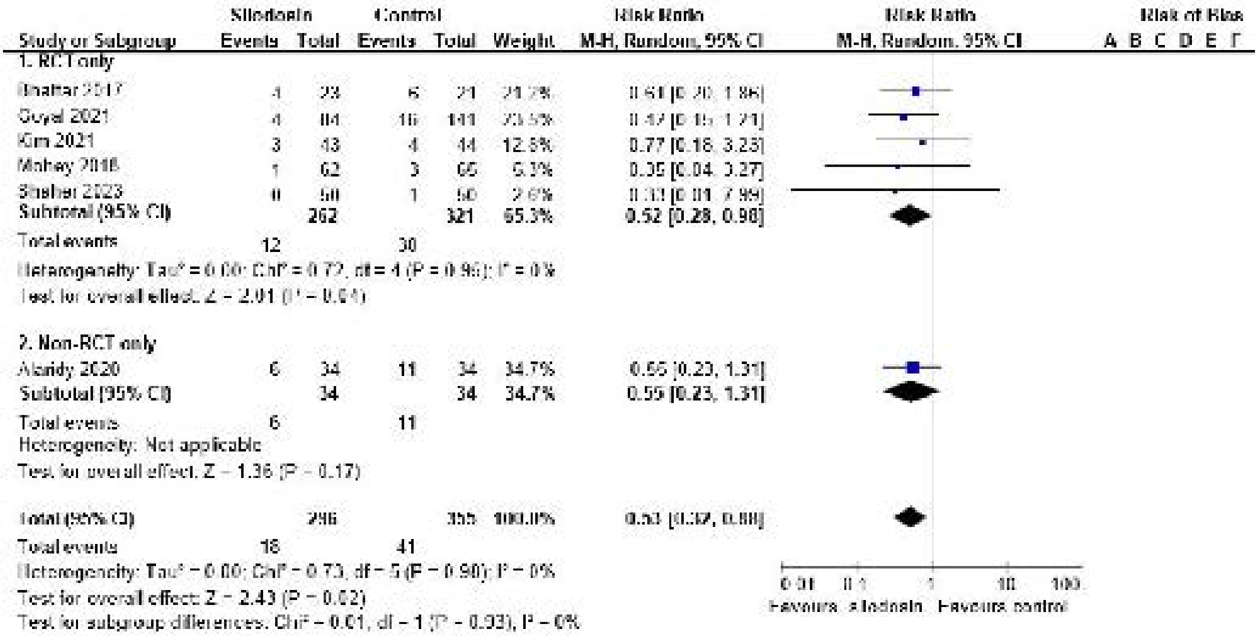

Subgroup Analyses - 10 days or more of silodosin preoperative therapy:

Figure S4 - Forrest plot: Ureteral wall injury (A) and Operative time (B).

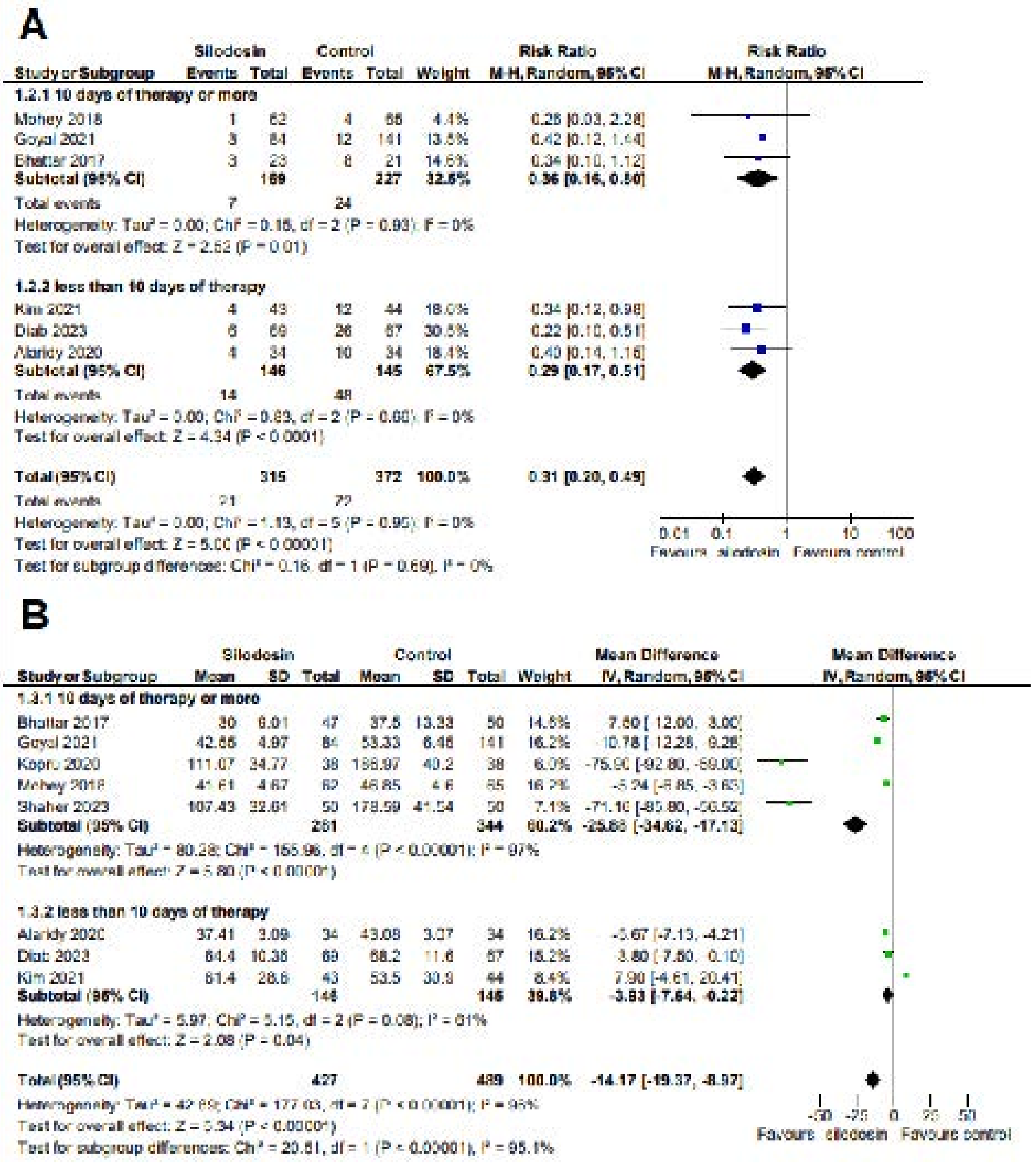

Subgroup Analyses - 10 days or more of silodosin preoperative therapy:

Figure S5 - Forrest plot: Need for ureteral dilation (A) and need for analgesia (B).

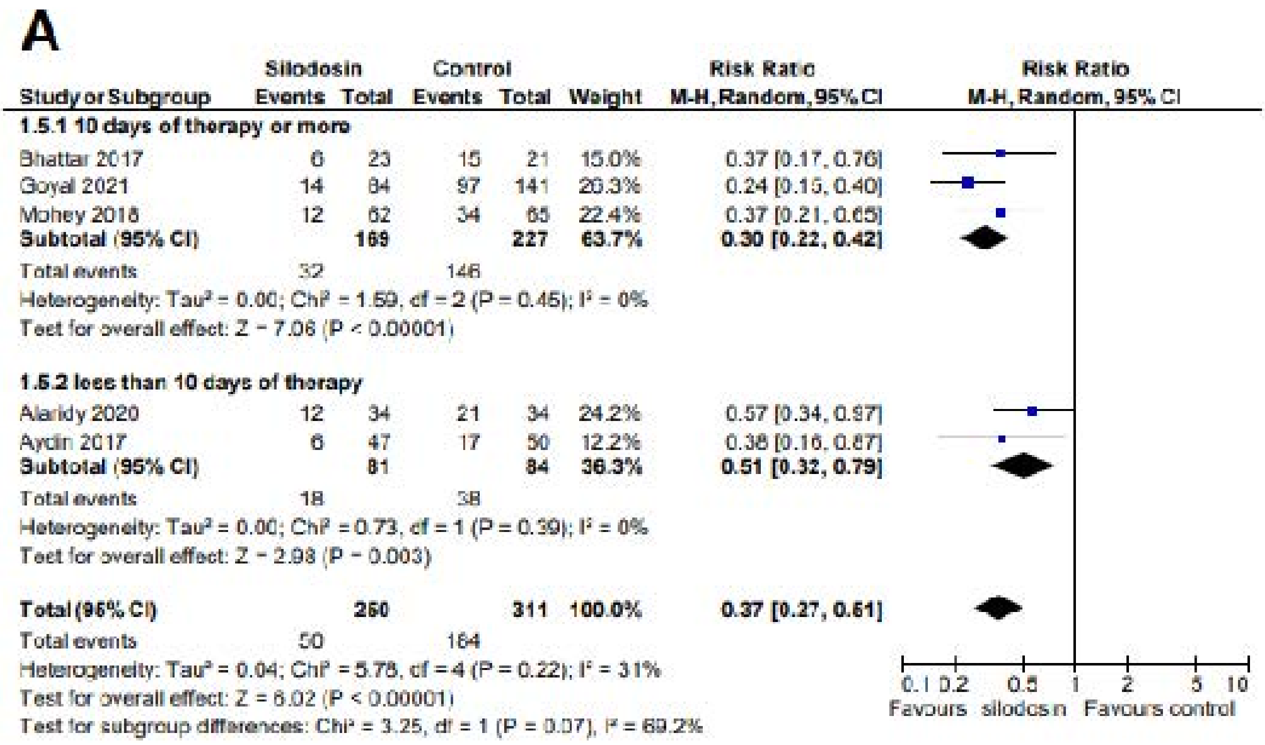

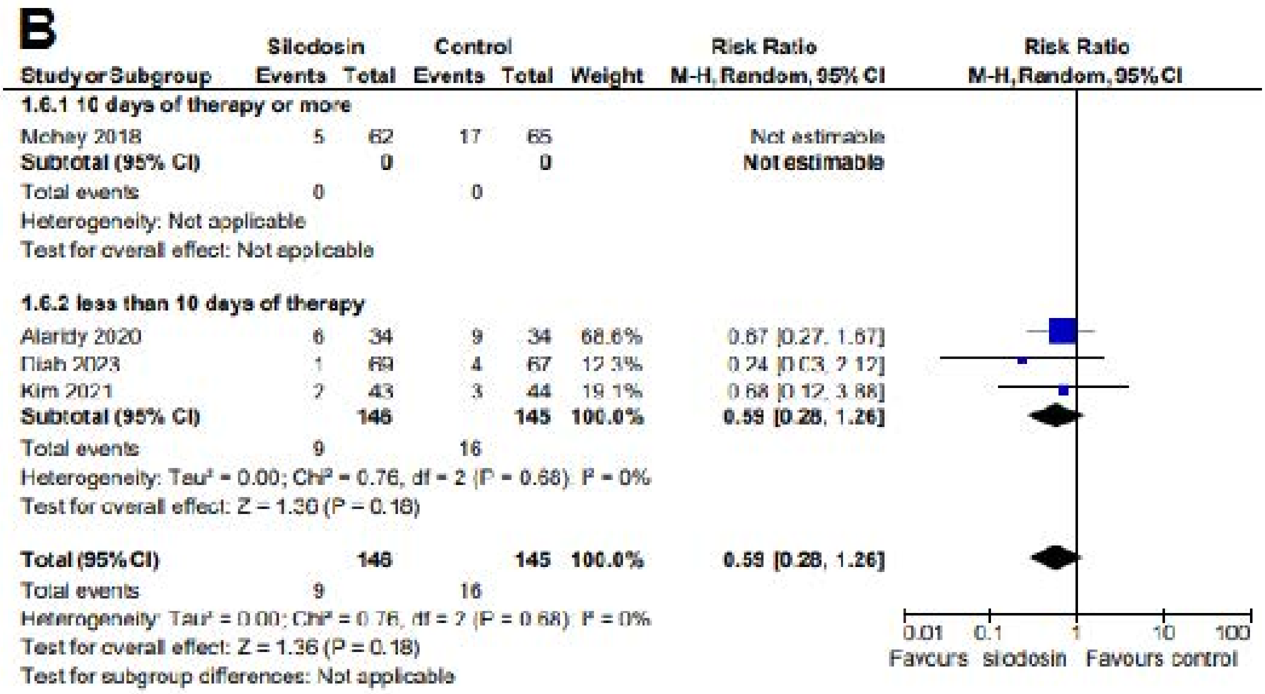

Subgroup Analyses - 10 days or more of silodosin preoperative therapy:

Figure S6 - Forrest plot: Post-operative fever (A) and haematuria (B).

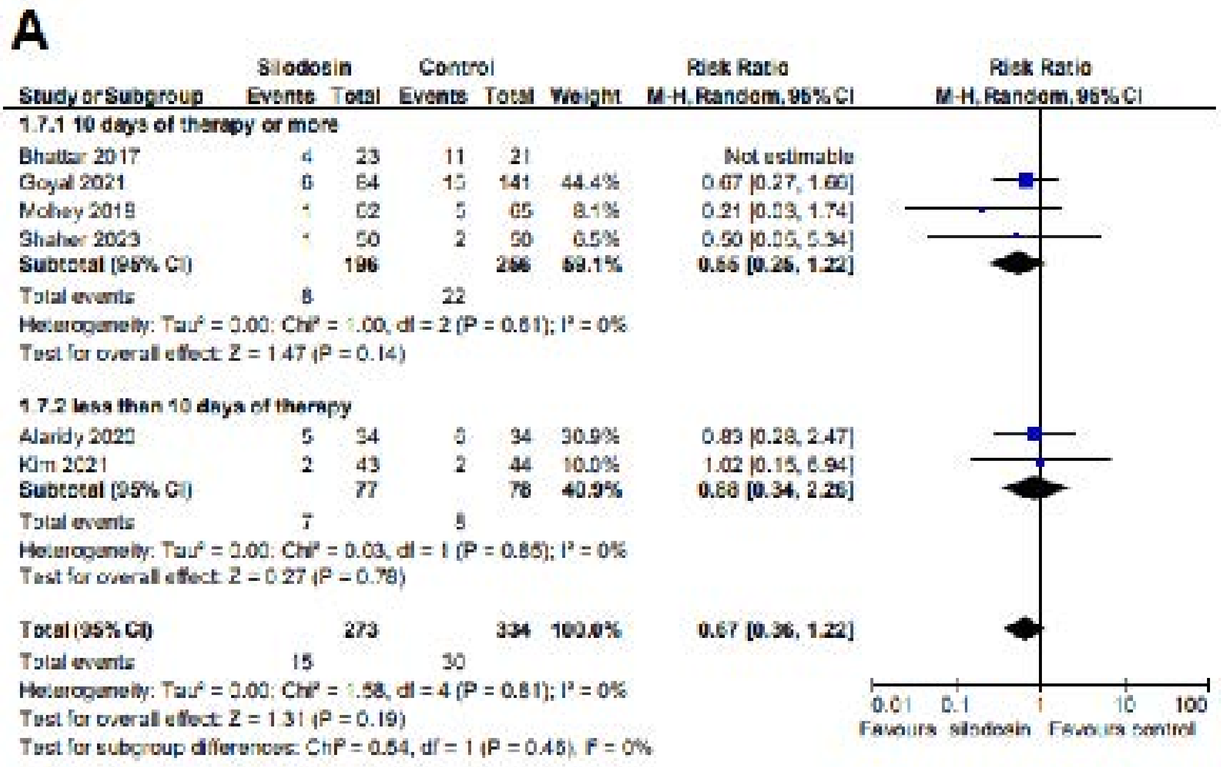

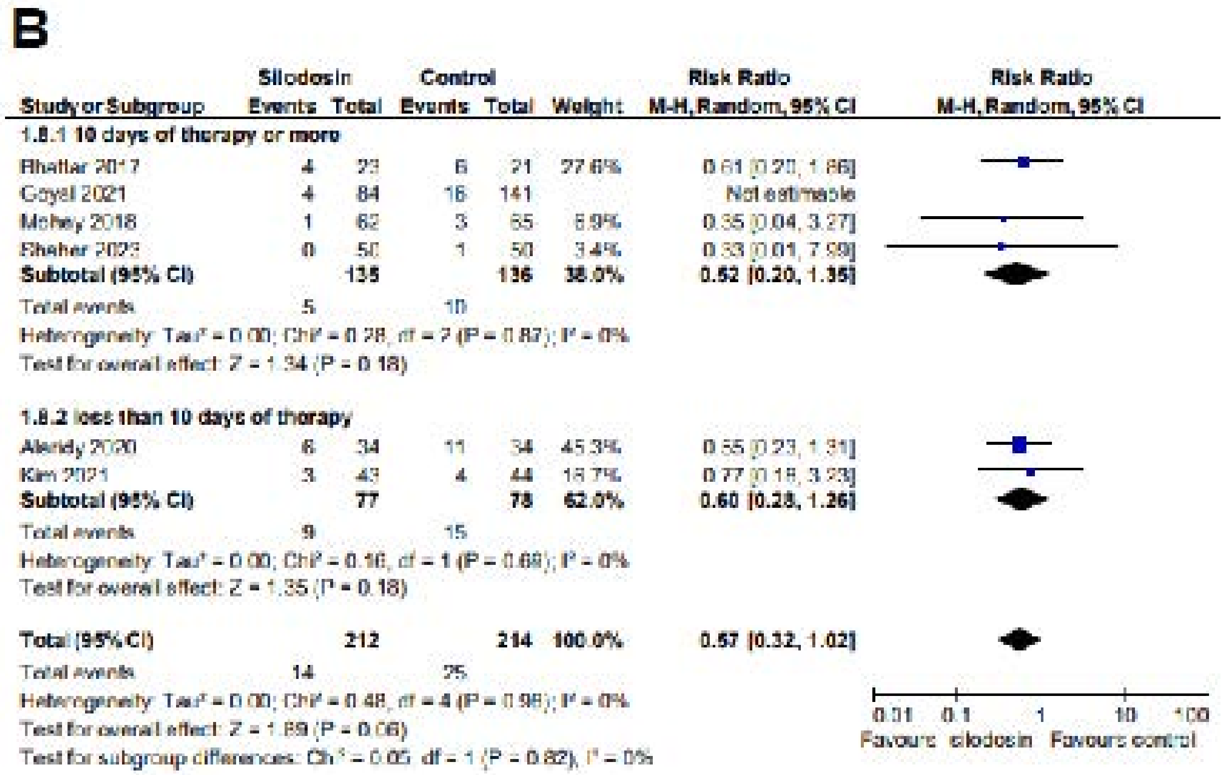

Subgroup Analyses - 10 days or more of silodosin preoperative therapy:

Figure S7 - Forrest plot: Stone-free rate.

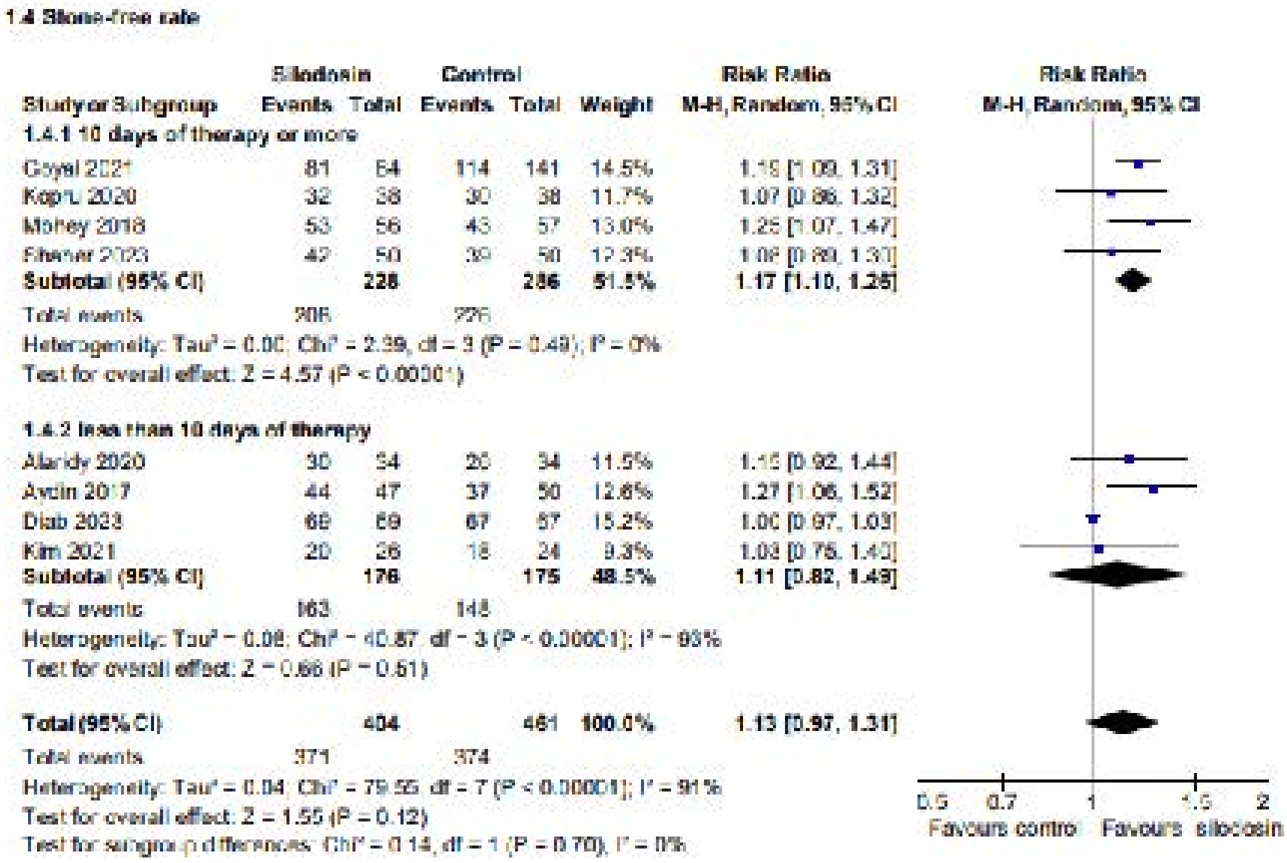

Subgroup Analyses - Different calculi location (proximal, distal, and mixed location):

Figure S8 - Forrest plot: Ureteral wall injury (A) and Operative time (B).

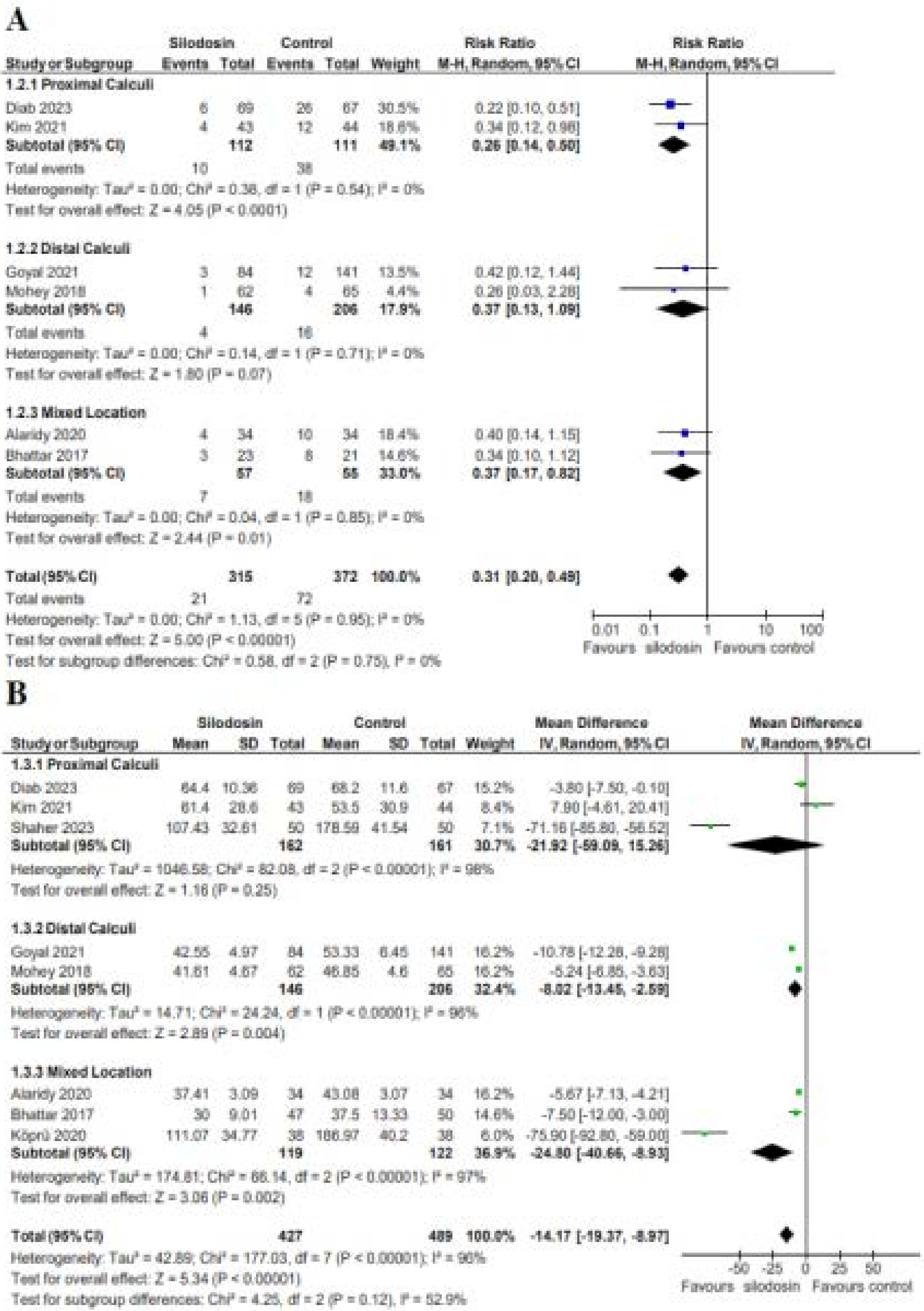

Subgroup Analyses - Different calculi location (proximal, distal, and mixed location):

Figure S9 - Forrest plot: Need for ureteral dilation (A) and need for analgesia (B).

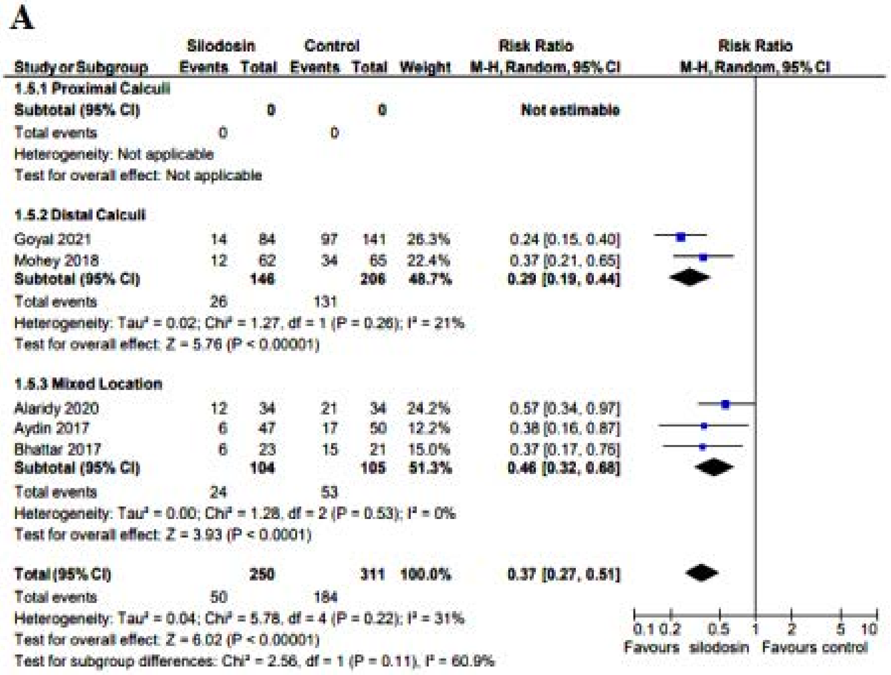

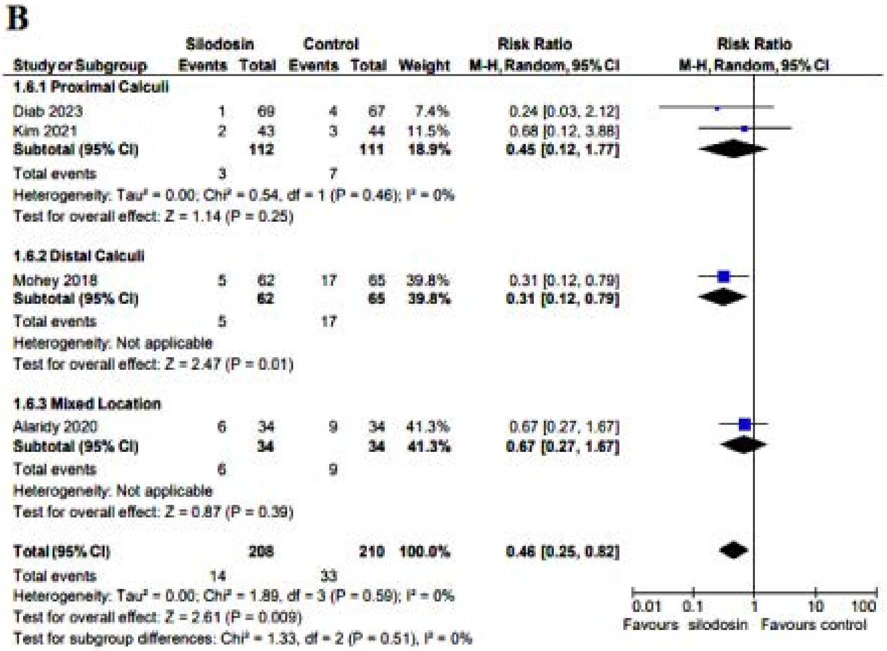

Subgroup Analyses - Different calculi location (proximal, distal, and mixed location):

Figure S10 - Forrest plot: Post-operative fever (A) and haematuria (B).

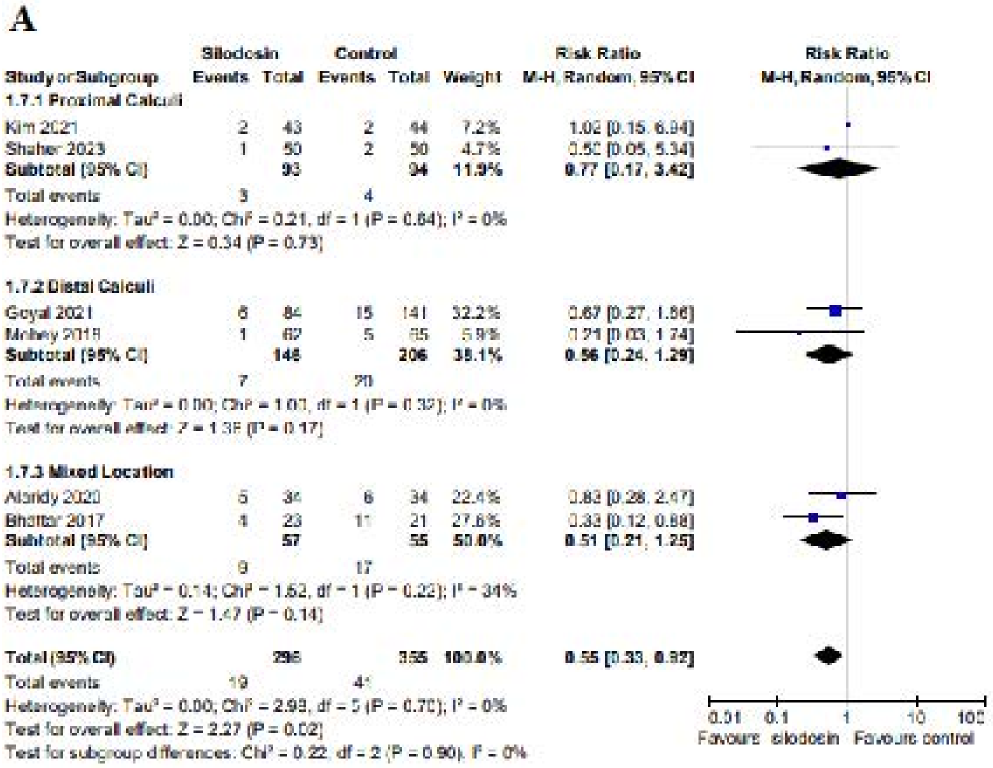

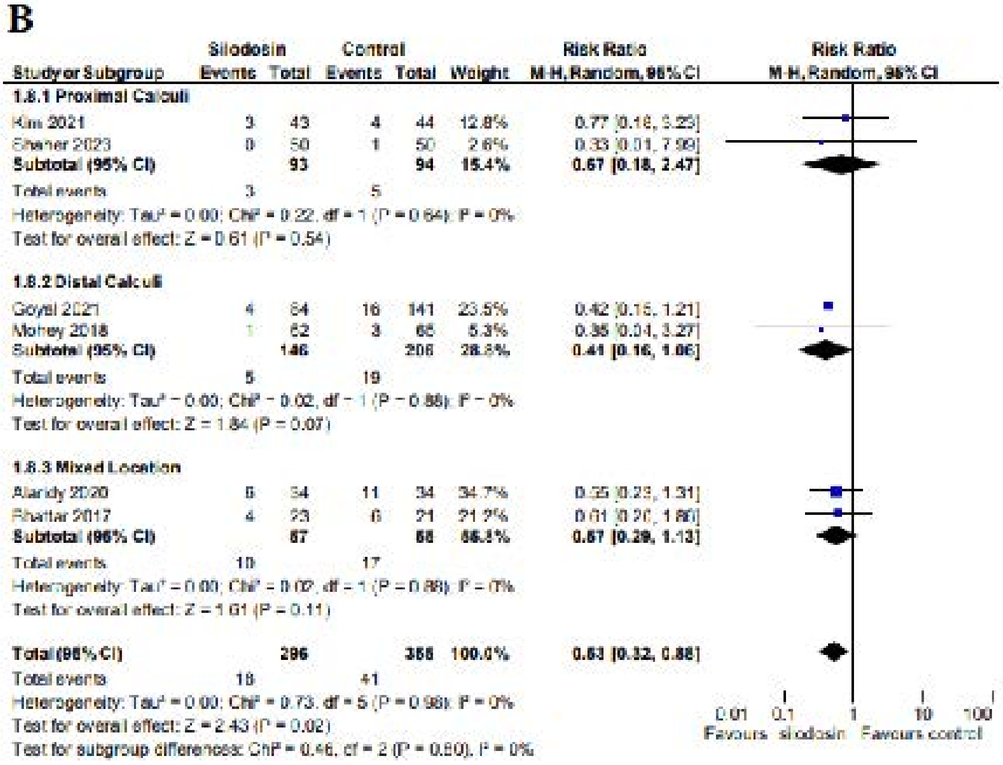

Subgroup Analyses - Different calculi location (proximal, distal, and mixed location):

Figure S11 - Forrest plot: Stone-free rate.

1.4 Stone-free rate

Study or Subgroup	Silodosin Events	Silodosin Total	Control Events	Control Total	Weight	Risk Ratio M-H, Random, 95% CI
**1.4.1 Proximal Calculi**						
Diab 2023	69	69	67	67	15.2%	1.00 [0.97, 1.03]
Kim 2021	20	26	18	24	9.3%	1.03 [0.75, 1.40]
Shaher 2023	42	50	39	50	12.3%	1.08 [0.89, 1.30]
**Subtotal (95% CI)**		**145**		**141**	**36.8%**	**1.02 [0.90, 1.16]**
Total events	131		124			
Heterogeneity: Tau² = 0.01; Chi² = 3.73, df = 2 (P = 0.16); I² = 46%						
Test for overall effect: Z = 0.35 (P = 0.73)						
**1.4.2 Distal Calculi**						
Goyal 2021	81	84	114	141	14.5%	1.19 [1.09, 1.31]
Mohey 2018	53	56	43	57	13.0%	1.25 [1.07, 1.47]
**Subtotal (95% CI)**		**140**		**198**	**27.5%**	**1.21 [1.12, 1.31]**
Total events	134		157			
Heterogeneity: Tau² = 0.00; Chi² = 0.31, df = 1 (P = 0.58); I² = 0%						
Test for overall effect: Z = 4.69 (P < 0.00001)						
**1.4.3 Mixed Location**						
Alaridy 2020	30	34	26	34	11.5%	1.15 [0.92, 1.44]
Aydin 2017	44	47	37	50	12.6%	1.27 [1.06, 1.52]
Köprü 2020	32	38	30	38	11.7%	1.07 [0.86, 1.32]
**Subtotal (95% CI)**		**119**		**122**	**35.7%**	**1.17 [1.04, 1.32]**
Total events	106		93			
Heterogeneity: Tau² = 0.00; Chi² = 1.45, df = 2 (P = 0.48); I² = 0%						
Test for overall effect: Z = 2.65 (P = 0.008)						
**Total (95% CI)**		**404**		**461**	**100.0%**	**1.13 [0.97, 1.31]**
Total events	371		374			
Heterogeneity: Tau² = 0.04; Chi² = 79.55, df = 7 (P < 0.00001); I² = 91%						
Test for overall effect: Z = 1.56 (P = 0.12)						
Test for subgroup differences: Chi² = 5.07, df = 2 (P = 0.08), I² = 60.6%						

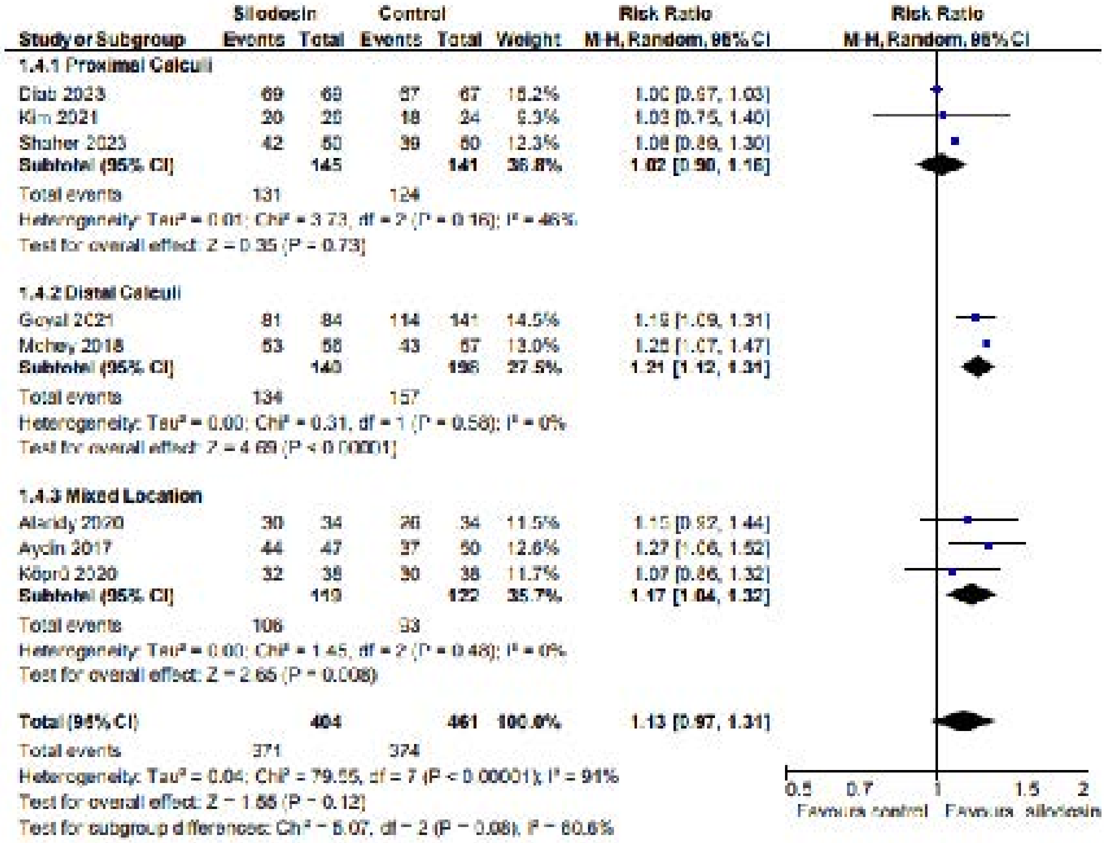

Figure S12 - PRISMA 2020 Checklist, Part 1.

Section and Topic	Item #	Checklist item	Location where item is reported
**TITLE**			
Title	1	Identify the report as a systematic review.	Title, page 1.
**ABSTRACT**			
Abstract	2	See the PRISMA 2020 for Abstracts checklist.	Abstract, page 2.
**INTRODUCTION**			
Rationale	3	Describe the rationale for the review in the context of existing knowledge.	Introduction, paragraph 2.
Objectives	4	Provide an explicit statement of the objective(s) or question(s) the review addresses.	Introduction, paragraph 3.
**METHODS**			
Eligibility criteria	5	Specify the inclusion and exclusion criteria for the review and how studies were grouped for the syntheses.	Methods –Inclusion and Exclusion Criteria.
Information sources	6	Specify all databases, registers, websites, organisations, reference lists and other sources searched or consulted to identify studies. Specify the date when each source was last searched or consulted.	Methods - Search Strategy.
Search strategy	7	Present the full search strategies for all databases, registers and websites, including any filters and limits used.	Supplementary Material – Table S1.
Selection process	8	Specify the methods used to decide whether a study met the inclusion criteria of the review, including how many reviewers screened each record and each report retrieved, whether they worked independently, and if applicable, details of automation tools used in the process.	Methods - Study Selection and Data Extraction.
Data collection process	9	Specify the methods used to collect data from reports, including how many reviewers collected data from each report, whether they worked independently, any processes for obtaining or confirming data from study investigators, and if applicable, details of automation tools used in the process.	Methods - Study Selection and Data Extraction.
Data items	10a	List and define all outcomes for which data were sought. Specify whether all results that were compatible with each outcome domain in each study were sought (e.g. for all measures, time points, analyses), and if not, the methods used to decide which results to collect.	Methods -Endpoints and Definitions.
	10b	List and define all other variables for which data were sought (e.g. participant and intervention characteristics, funding sources). Describe any assumptions made about any missing or unclear information.	Results – Table 1. Supplementary Material – Table S2, Table S3.
Study risk of bias assessment	11	Specify the methods used to assess risk of bias in the included studies, including details of the tool(s) used, how many reviewers assessed each study and whether they worked independently, and if applicable, details of automation tools used in the process.	Methods - Quality Assessment.
Effect measures	12	Specify for each outcome the effect measure(s) (e.g. risk ratio, mean difference) used in the synthesis or presentation of results.	Methods - Statistical Analysis.
Synthesis methods	13a	Describe the processes used to decide which studies were eligible for each synthesis (e.g. tabulating the study intervention characteristics and comparing against the planned groups for each synthesis (item #5)).	Methods - Endpoints and Definitions.
	13b	Describe any methods required to prepare the data for presentation or synthesis, such as handling of missing summary statistics, or data conversions.	Methods - Statistical Analysis; Results – Table 1.
	13c	Describe any methods used to tabulate or visually display results of individual studies and syntheses.	Methods –

Figure S13 - PRISMA 2020 Checklist, Part 2.

Section and Topic	Item #	Checklist item	Location where item is reported
			Statistical Analysis.
	13d	Describe any methods used to synthesize results and provide a rationale for the choice(s). If meta-analysis was performed, describe the model(s), method(s) to identify the presence and extent of statistical heterogeneity, and software package(s) used.	Methods - Statistical Analysis.
	13e	Describe any methods used to explore possible causes of heterogeneity among study results (e.g. subgroup analysis, meta-regression).	Methods - Statistical Analysis.
	13f	Describe any sensitivity analyses conducted to assess robustness of the synthesized results.	Methods - Statistical Analysis.
Reporting bias assessment	14	Describe any methods used to assess risk of bias due to missing results in a synthesis (arising from reporting biases).	Methods – Quality Assessment
Certainty assessment	15	Describe any methods used to assess certainty (or confidence) in the body of evidence for an outcome.	Methods - Statistical Analysis.
**RESULTS**			
Study selection	16a	Describe the results of the search and selection process, from the number of records identified in the search to the number of studies included in the review, ideally using a flow diagram.	Results – Figure 1.
	16b	Cite studies that might appear to meet the inclusion criteria, but which were excluded, and explain why they were excluded.	Results – Figure 1.
Study characteristics	17	Cite each included study and present its characteristics.	Results – Table 1.
Risk of bias in studies	18	Present assessments of risk of bias for each included study.	Supplementary Material – Figure S1.
Results of individual studies	19	For all outcomes, present, for each study: (a) summary statistics for each group (where appropriate) and (b) an effect estimate and its precision (e.g. confidence/credible interval), ideally using structured tables or plots.	Results – Figure 2; Supplementary Material – Figure S2-S7.
Results of syntheses	20a	For each synthesis, briefly summarise the characteristics and risk of bias among contributing studies.	Results - Quality Assessment.
	20b	Present results of all statistical syntheses conducted. If meta-analysis was done, present for each the summary estimate and its precision (e.g. confidence/credible interval) and measures of statistical heterogeneity. If comparing groups, describe the direction of the effect.	Results – Figure 2; Supplementary Material – Figure S2-S7.
	20c	Present results of all investigations of possible causes of heterogeneity among study results.	Results -Endpoints Pooled analyses; Discussion, paragraph 6 and 7.
	20d	Present results of all sensitivity analyses conducted to assess the robustness of the synthesized results.	Results - Sensitivity analyses.
Reporting biases	21	Present assessments of risk of bias due to missing results (arising from reporting biases) for each synthesis assessed.	Results - Quality Assessment.
Certainty of	22	Present assessments of certainty (or confidence) in the body of evidence for each outcome assessed.	Results - Endpoints

Figure S14 - PRISMA 2020 Checklist, Part 3.

Section and Topic	Item #	Checklist item	Location where item is reported
evidence			Pooled analysis.
**DISCUSSION**			
Discussion	23a	Provide a general interpretation of the results in the context of other evidence.	Discussion, paragraph 2, 3, 4, 5, 7 and 8.
	23b	Discuss any limitations of the evidence included in the review.	Discussion – Limitation.
	23c	Discuss any limitations of the review processes used.	Discussion – Limitation.
	23d	Discuss implications of the results for practice, policy, and future research.	Discussion, paragraph 1. Conclusion.
**OTHER INFORMATION**			
Registration and protocol	24a	Provide registration information for the review, including register name and registration number, or state that the review was not registered.	Methods, paragraph 1.
	24b	Indicate where the review protocol can be accessed, or state that a protocol was not prepared.	Methods, paragraph 1.
	24c	Describe and explain any amendments to information provided at registration or in the protocol.	Acknowledgements.
Support	25	Describe sources of financial or non-financial support for the review, and the role of the funders or sponsors in the review.	Acknowledgements.
Competing interests	26	Declare any competing interests of review authors.	Acknowledgements.
Availability of data, code and other materials	27	Report which of the following are publicly available and where they can be found: template data collection forms; data extracted from included studies; data used for all analyses; analytic code; any other materials used in the review.	Not applicable.

From: Page MJ, McKenzie JE, Bossuyt PM, Boutron I, Hoffmann TC, Mulrow CD, et al. The PRISMA 2020 statement: an updated guideline for reporting systematic reviews. BMJ 2021;372:n71. doi: 10.1136/bmj.n71.
This work is licensed under CC BY 4.0. To view a copy of this license, visit https://creativecommons.org/licenses/by/4.0/

Table S1 - Detailed search strategy according to each database.

Database	Search strategy
PubMed/ MEDLINE	("ureteral stones" OR "ureteral calculi"[Mesh] OR "ureteral stone" OR "ureteral calculi" OR "ureterolithiasis" OR "ureteric stones" OR "ureteric calculi" OR "ureteric stone" OR "ureteric calculi" OR "ureteroscopy" OR "ureteroscopic" OR "ureterorenoscopy" OR "ureteral access") AND ("silodosin")
Embase	('ureteral stones' OR 'ureteral calculi' OR 'ureteral stone' OR 'ureteral calculi' OR 'ureterolithiasis' OR 'ureteric stones' OR 'ureteric calculi' OR 'ureteric stone' OR 'ureteric calculi' OR 'ureteroscopy' OR 'ureteroscopic' OR 'ureterorenoscopy' OR 'ureteral access') AND ('silodosin')
Cochrane	("ureteral stones" OR "ureteral calculi"[Mesh] OR "ureteral stone" OR "ureteral calculi" OR "ureterolithiasis" OR "ureteric stones" OR "ureteric calculi" OR "ureteric stone" OR "ureteric calculi" OR "ureteroscopy" OR "ureteroscopic" OR "ureterorenoscopy" OR "ureteral access") AND ("silodosin")

Table S2 - Extended baseline characteristics of the included studies.

Study, year	Study Design	Type of Control	Follow-up (months)	Time of therapy (days)	Baseline Population Size, No. Silodosin Control	Age, years (mean ± SD) Silodosin Control	Male, No. (%) Silodosin Control	BMI, kg/mm² (mean ± SD) Silodosin Control	Stone size, mm (mean ± SD) Silodosin Control	Location of ureteral calculi (upper/ middle/ lower) Silodosin Control	Stent Placement, No. (%) Silodosin Control	Type of procedure (both groups)	Ureteral access sheat (both groups)	Surgeon experience (both groups)	Anatomical abnormality (both groups)	History of ipsilateral ureteric surgery (both groups)	Infection (both groups)	Stone surface, mm² (median ± SD) Silodosin Control	Stone density, Hounsfeld unit (mean ± SD) Silodosin Control
Alaridy et al., 2020 (2)	Non-RCT	Placebo	1	7	34<br>34	33.29 ± 9.51<br>34.60 ± 12.01	25 (73.52)<br>25 73.52)	NA	10.35 ± 2.38<br>10.41 ± 2.43	3/6/25<br>3/6/25	NA	Semi-rigid	NA	Senior urologist	Excluded	Excluded	Excluded	NA	NA
Aydin et al., 2017 (11)	RCT	No pretreatment	1	3	47<br>5	43.00 ± 14.29*<br>37.50 ± 12.50*	32 (68.08)<br>33 (66.00)	NA	NA	12/9/26<br>8/12/30	NA	Semi-rigid	NA	NA	NA	Excluded	Excluded	38 (NA)<br>35.5 (NA)	NA
Bhattar et al., 2017 (12)	RCT	Placebo	NA	14	23<br>21	35.52 ± 11.00<br>33.22 ± 10.07	15 (65.21)<br>15 (71.42)	23.34<br>34.10	9.14 ± 1.52<br>9.74 ± 1.98	5/4/14<br>6/3/12	23 (100.00)<br>21 (100.00)	NA	NA	Senior urologist	Excluded	Excluded	Excluded	NA	NA
Diab et al., 2023 (1)	RCT	Placebo	3	7	69<br>67	41.40 ± 14.26<br>42.40 ± 15.44	38 (46.37)<br>41 (61.19)	26.90 ± 3.79,<br>27.30 ± 3.97	12.50 ± 3.91<br>13.00 ± 3.71	70/0/0<br>70/0/0	69 (100.00)<br>67 (100.00)	Flexible	Yes	Senior urologist	Excluded	Excluded	Excluded	NA	1022 ± 259.6<br>1008.7 ± 266.4
Goyal et al., 2021 (13)	RCT	Placebo	0.5	10	84<br>141	39.28 ± 8.25<br>38.22 ± 8.34	53 (63.19)<br>86 (60.99)	27.75 ± 2.22<br>27.46 ± 2.29	8.77 ± 4.12<br>8.53 ± 0.49	0/0/84<br>0/0/93	NA	Semi-rigid	NA	Senior urologist	Excluded	Excluded	NA	NA	NA
Kim et al., 2021 (14)	RCT	No pretreatment	3	3	43<br>44	48.50 ± 11.60,<br>45.80 ± 13.80	29 (67.44)<br>23 (52.27)	26.80± 4.90,<br>25.20 ± 3.30	8.86 ± 3.60<br>8.68 ± 5.07	50/0/0<br>50/0/0	43 (100.00)<br>44 (100.00)	Flexible	Yes	Senior urologist	Excluded	Excluded	NA	NA	589 ± 290<br>532 ± 325
Köprü et al., 2020 (15)	RCT	No pretreatment	3	10	38<br>38	45.41 ± 12.88*,<br>46.52 ± 14.52*	30 (78.94)<br>23 (60.52)	NA	19.02 ± 5.90<br>17.94 ± 4.60	2/8/6<br>2/6/7	21 (55.26)<br>23 (60.52)	Flexible	Yes	NA	NA	NA	NA	NA	NA
Mohey et al., 2018 (16)	RCT	Placebo	1	10	62<br>65	38.27 ± 9.37,<br>39.67 ± 9.54	39 (62.90)<br>39 (60.00)	27.55 ± 2.28<br>27.80 ± 3.50	12.60 ± 1.25<br>12.90 ± 1.29	0/0/62<br>0/0/65	62 (100.00)<br>65 (100.00)	Semi-rigid	NA	Senior urologist	Excluded	Excluded	NA	NA	907.66 ± 208.52<br>898.97 ± 212.04
Shaher et al., 2023 (17)	RCT	Placebo	1	10	50<br>50	44.65 ± 10.13<br>45.37 ± 12.78	37 (74.00)<br>30 (60.00)	26.12 ± 2.63<br>26.34 ± 2.74	18.33 ± 5.17<br>17.61 ± 4.25	11/0/0<br>8/0/0	26 (52.00)<br>30 (60.00)	Flexible	Yes	NA	Excluded	Excluded	NA	NA	837.64 ± 234.3<br>819.5 ± 240.1

BMI = Body Mass Index; RCT = Randomised controlled trial; NA = Not available

* Mean and standard deviation (SD) estimated from median and interquartile range or median and range
